# Synthesis and Characterization of Lithium Mining Waste and Metakaolin-Based Geopolymers

**DOI:** 10.1021/acsomega.5c11473

**Published:** 2026-05-06

**Authors:** Vinícius F. C. Sampaio, Ana Gabriela Fernandes, Jeniffer Fernandes, Suellen Almeida, Lucas Lorenzini, Rochel Montero Lago, Ana Paula C Teixeira

**Affiliations:** Departamento de Química, ICEx, 28114 Universidade Federal de Minas Gerais (UFMG), Av. Antônio Carlos, 6627 Pampulha, Belo Horizonte, MG, Brazil

## Abstract

The growing demand for lithium has driven aggressive extraction activities, often associated with several environmental challenges, such as tailings dams. It is estimated that for every ton of lithium carbonate produced, approximately 10 tons of refinery waste are generated. In this scenario, geopolymers have emerged as a promising alternative for recycling large volumes of waste into high-value, technologically advanced products. This study presents a detailed investigation into the influence of lithium mining waste (LiMW) on the mechanical and chemical properties of metakaolin-based geopolymers. The LiMW is mainly composed of albite, quartz, and muscovite. Two types of metakaolin were employed as aluminosilicate sources, and varying proportions of alkaline solution were used to synthesize geopolymers incorporating 0 to 60% LiMW. The incorporation of mining waste, up to an adequate proportion, significantly enhanced the compressive strength of both syntheses. The best compressive strength was achieved by incorporating 40% of mining waste in both syntheses. The geopolymer produced with Sulfal Metakaolin (MKS) achieved 54.1 MPa, while the geopolymer synthesized with Metakaolin of Brazil (MKB) reached 32.4 MPa after 7 days of curing. The optimal liquid-to-solid ratios reached using MKS and MKB were 0.34 and 0.30, respectively. XRD, FT-IR, ^27^Al and ^29^Si NMR, and SEM/EDS characterized the resulting geopolymers of both syntheses. The results obtained in this work are comparable to or even exceed those achieved with Portland cement, highlighting the applicability of LiMW as a viable component in geopolymeric binders, especially in materials applied to the civil construction sector.

## Graphical abstract

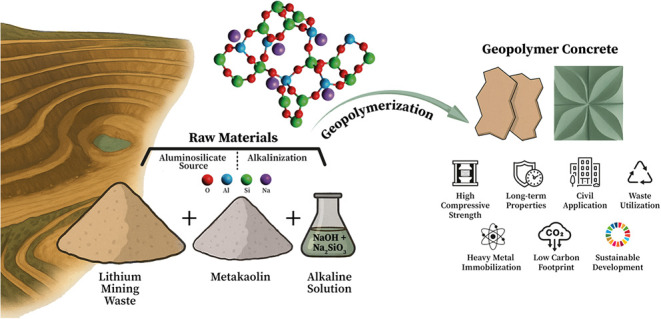

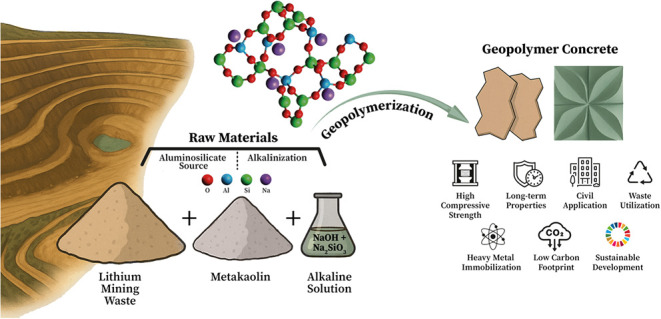

## 1 Introduction

The global demand for lithium has increased significantly over the years, driven by its use in emerging technologies, such as rechargeable batteries for electric vehicles and electronic devices, and the pursuit of sustainable solutions to conventional fossil fuel-based energy sources.^1–3^ In 2024, global lithium production reached approximately 240,000 tons, an 18% increase compared to the previous year. Australia, Chile, and China were the largest producers, with Brazil accounting for about 4% of this output.^4^ Lithium is used to develop batteries and energy storage systems, as well as in the production of ceramics, glass, alloys, thickeners, and pharmaceutical products.^3,5,6^

Lithium is associated with numerous minerals found in igneous rocks, such as granite and pegmatites, with spodumene being one of the most common.^6^ The intensification of lithium extraction activities is associated with several environmental challenges, particularly related to the generation of solid waste. One of the beneficiation processes for obtaining lithium carbonate, for example, involves high-temperature roasting followed by sulfuric acid leaching.^5^ It is estimated that for every ton of lithium carbonate produced, approximately 10 tons of refinery waste are generated, resulting in over 5 million tons of this byproduct annually.^5^ Due to the mineralogical nature of spodumene, the waste is commonly disposed of in tailings dams, forming deposits predominantly composed of Al_2_O_3_ and SiO_2_.^2^

In this context, geopolymers have emerged as a promising alternative. First developed by Davidovits in the 1970s, geopolymers are a class of inorganic materials synthesized through the reaction of aluminosilicate-rich precursors in an alkaline or acidic medium.^7,8^ Although the geopolymerization mechanism is not fully understood, the conventional process is generally described in three main stages: (1) dissolution of alumina and silica in an alkaline medium, (2) condensation of silicate and aluminate species to form Si-rich and Al-rich gel, and (3) polycondensation of these gels to develop the three-dimensional geopolymer network.^9–12^ Geopolymers have demonstrated attractive properties, especially for civil construction applications, due to their high early compressive strength, fast hardening, high thermal stability, low permeability, and good resistance to chemical attack. Moreover, the optimal design of geopolymers requires less energy consumption and can result in a lower CO_2_ footprint than traditional Portland cement materials.^12,13^

Currently, a great number of industrial byproducts have been explored as aluminosilicate raw materials or aggregates in geopolymer synthesis.^14–18^ Fly ash,^19–21^ bottom ash,^22^ red mud,^23,24^ rice hull and husk ashes,^25,26^ blast furnace slag,^25^ iron ore tailings,^14,15,27^ steel slag,^28^ lithium slag,^29,30^ tungsten waste,^31^ and construction solid waste^32,33^ are examples of waste materials that have been applied in geopolymer and concrete research. However, the synthesis of geopolymers is a complex process strongly dependent on experimental conditions. Several factors, such as the chemical composition of the precursors, the degree of amorphization or crystallinity of the aluminosilicate source, the molar ratio of the alkaline reagent (SiO_2_/R_2_O, where R can be Na^+^ or K^+^), and the liquid-to-solid ratio, directly influence the dissolution rate of alumina- and silica-rich species. These variables play a decisive role in the subsequent condensation steps, the rearrangement of sialate units, and the final formation of the three-dimensional geopolymeric structure.^10–12,34–37^

Under these circumstances, this research focuses on a detailed and systematic investigation of the compressive strength and physicochemical properties of geopolymers synthesized with varying proportions of lithium mining waste (LiMW), using two different aluminosilicate sources (metakaolin) and distinct alkaline reactant conditions. It is also important to emphasize that this study assessed the use of a low-grade commercial metakaolin and demonstrated the feasibility of producing high-quality, cost-effective geopolymers, thereby supporting their potential for widespread application within the civil construction sector.

The results of this work not only contribute to the development of new sustainable technologies capable of minimizing the environmental impacts of mineral extraction and transforming large volumes of waste into high-value products, but also offer an alternative to reduce the exploitation of natural resources and address environmental issues associated with landfilling, tailing dams, and other waste disposal practices, while promoting the development of more sustainable and accessible geopolymeric materials.

## 2 Experimental Details

### 2.1 Raw Materials

Two local types of metakaolin (MK) were used as aluminosilicate precursors: Sulfal Metakaolin (MKS) and Metakaolin of Brazil. MKS was obtained by thermally treating Sulfal kaolin at 800 °C, under an air atmosphere, with a heating rate of 20 °C/min and a dwell time of 4 h at the maximum temperature. Otherwise, MKB was commercially supplied and does not require any additional process before use. The chemical composition (determined by XRF analysis), median particle size, specific surface area, and loss of ignition of both aluminosilicate sources are presented in Table 1 and Figure S1.

**Table 1:** Chemical Composition and Particle Size of the Metakaolins

Sulfal Metakaolin		
Composition	Oxide	wt %
	SiO_2_	52.40
	Al_2_O_3_	43.37
	MgO	2.12
	Fe_2_O_3_	1.05
	TiO_2_	0.51
	K_2_O	0.29
Si/Al molar ratio		1.07
Particle size (μm)	*d*_10_	2.44
	*d*_50_	14.13
	*d*_90_	45.27
Median particle size (μm)		19.56
Specific surface area		15.64 m^2^/g
Loss on ignition at 900 °C		0.10%

Metakaolin of Brazil		
Composition	Oxide	wt %
	SiO_2_	56.90
	Al_2_O_3_	34.10
	Fe_2_O_3_	1.78
	TiO_2_	1.36
	K_2_O	1.25
	MgO	0.26
Si/Al molar ratio		1.48
Particle size (μm)	*d*_10_	3.13
	*d*_50_	22.89
	*d*_90_	62.98
Median particle size (μm)		28.90
Specific surface area		19.19 m^2^/g
Loss on ignition at 900 °C		4.30%

AMG Brazil S.A. provided the lithium mining waste (LiMW). The material was collected from their waste storage piles, air-dried at room temperature, and sifted to remove large particles and impurities, such as small rocks. Table 2 presents its chemical composition (determined by XRF analysis), median particle size, specific surface area and loss of ignition. XRF analysis confirms that no residual Li content is present in the waste used in this study.

**Table 2:** Chemical Composition and Particle Size of the LiMW

Composition	Oxide	wt %
	SiO_2_	71.47
	Al_2_O_3_	16.21
	K_2_O	1.98
	TiO_2_	0.83
	Fe_2_O_3_	0.32
	CaO	0.26
	BaO	0.23
	PbO	0.19
	Y_2_O_3_	0.15
Particle size (μm)	*d*_10_	42.23
	*d*_50_	130.23
	*d*_90_	256.56
Median particle size (μm)		141.98
Specific surface area		0.22 m^2^/g
Loss on ignition at 900 °C		0.65%

Una-Prosil Brazil commercially supplied the basic solutions used in this research. The sodium silicate contained 33.2 wt % SiO_2_, 15.1 wt % Na_2_O, and 51.7 wt % H_2_O, and the sodium hydroxide solution (50% m/v) had a density of 1.6 g/cm^3^. The desired SiO_2_/Na_2_O molar ratio of 1.8 was achieved by blending the alkaline solutions. This choice was not arbitrary; it was based on a consistent synthesis method developed by Davidovits to produce geopolymers with high chemical stability, reproducibility, mechanical strength, and long-term durability.^38^

### 2.2 Geopolymer Synthesis

This research investigates two distinct geopolymer synthesis methods, each with a specific objective: to evaluate the effect of LiMW, sodium silicate molar ratio, metakaolin proportion, Si/Al ratio, and liquid-to-solid ratio on compressive strength and chemical properties. Table 3 shows the geopolymers formulations denoted by codes as MK­[*x*]·Li­[*y*], where “*x*” refers to the type of metakaolin, and “*y*” indicates the amount of LiMW used.

**Table 3:** Geopolymer Formulations with Varying Proportions of LiMW, MK, and AS (wt %)^a^

Geopolymers	LiMW	MK	AS	Si/Al molar ratio	Liquid-to-solid ratio
MKS.LiMW[0]	0	40.16	59.84	2.07	0.91
MKS.LiMW[10]	10	36.14	53.86	2.24	0.71
MKS.LiMW[20]	20	32.13	47.87	2.42	0.56
MKS.LiMW[30]	30	28.11	41.89	2.59	0.44
MKS.LiMW[40]	40	24.10	35.90	2.77	0.34
MKS.LiMW[50]	50	20.08	29.92	2.95	0.26
MKS.LiMW[60]	60	16.06	23.94	3.13	0.23
MKB.LiMW[0]	0	46.08	53.92	2.48	0.72
MKB.LiMW[10]	10	41.47	48.53	2.62	0.58
MKB.LiMW[20]	20	36.87	43.13	2.77	0.46
MKB.LiMW[30]	30	32.26	37.74	2.91	0.38
MKB.LiMW[40]	40	27.65	32.35	3.05	0.30
MKB.LiMW[50]	50	23.04	26.96	3.20	0.26
MKB.LiMW[60]	60	18.43	21.57	3.34	0.23

a AS: Alkaline solution.

The geopolymeric materials were synthesized by mechanically mixing the alkaline solution at a low speed for 10 min using a planetary mixer. Metakaolin was then added and mixed for an additional 10 min—5 min at low speed and 5 min at high speed. In formulations containing LiMW, this material was added after the metakaolin and mixed at high speed for 5 min, totaling 1.6 kg of geopolymer paste per batch. Five cylindrical samples with 100 mm of height and 50 mm of diameter were prepared for the compressive strength tests. All samples were stored in sealed plastic for 24 h, then demolded and cured at ambient temperature for 7, 14, and 28 days.

### 2.3 Compressive Strength Tests

After 7 days of curing, the dimensions of five replicate cylindrical samples were measured before undergoing compressive strength tests. Only geopolymer formulations exhibiting high compressive strength values were further evaluated after 14 and 28 days of curing. The tests were performed using a manually operated hydraulic press, and the force was applied via a hand-operated hydraulic pump. Compressive strength results were recorded in kgf/cm^2^ and converted to MPa following NBR 7222.

### 2.4 Physicochemical Characterization

The chemical composition of the raw materials and the selected geopolymer formulations was evaluated using the following techniques:

- **X-ray fluorescence (XRF)** using an energy-dispersive X-ray fluorescence (EDXRF) spectrometer, the ARL QUANT’X-Thermo Scientific, with a rhodium tube (50 W). The sample analysis was performed under a vacuum. Data acquisition was carried out using the WINTRACE software, and the data were processed with the Uniquant software, which is based on fundamental parameters.
- **X-ray diffraction (XRD)** using an Empyrean X-ray diffractometer from PANalytical. The analysis was conducted in continuous scan mode, covering an angular range of 3.03° to 89.97° 2θ with a step size of 0.06° and a scan time of 1 s per step. A fixed divergence slit of 0.4354° was used, along with a receiving slit of 0.19 mm. The measurements were performed at room temperature (25 °C) using a copper (Cu) anode as the X-ray source, which generated K-α radiation with wavelengths of 1.54060 Å (Kα1) and 1.54443 Å (Kα2).
- **Fourier transform infrared spectroscopy with attenuated total reflectance (ATR-FTIR)** using a Bruker α spectrometer equipped with the α-T Universal Sampling Module, operating in transmission mode. The spectra were recorded with a resolution of 4 cm^–1^ and averaged over 64 scans. A background spectrum was collected under the same conditions before each sample measurement to guarantee data accuracy. All spectral acquisition and processing were performed using the OPUS software.
- **Thermogravimetric analysis (TGA)** using a Shimadzu Simultaneous TGA/DTA Analyzer DTG-60H at the Laboratory of Environmental Technologies (GruTAm), Department of Chemistry, UFMG. The analyses were conducted in an alumina crucible under a flowing air atmosphere with a flow rate of 50 mL^–1^. The furnace was programmed with a heating rate of 10 °C·min^–1^, starting from room temperature and reaching up to 900 °C. The sample weights ranged from 1 to 2 mg.
- **Physisorption analysis** of N_2_ was performed using Autosorb iQ equipment (Quantachrome Instruments, USA) at −196 °C in the relative pressure range from 0.001 to 1.0 atm. The metakaolin samples were previously degassed at 200 °C for 6 h under vacuum conditions. The data obtained were analyzed using ASiQwin software version 5.21, and the BET method (Brunauer–Emmett–Teller) was used to estimate the specific surface area (SSA).
- **Particle size distribution (PSD)** was measured using a CILAS laser granulometer, model 1190 Particle Size Analyzer, with a measurement range from 0.04 to 2500 μm, located at the Nuclear Fuel Laboratory of CDTN/CNEN. The analysis was performed in triplicate.
- **Scanning electron microscopy (SEM)** was performed using a FEI Quanta 200 FEG scanning electron microscope, equipped with a field emission gun (FEG) that allows stable operation at accelerating voltages between 2 kV and 30 kV.
- **Energy dispersive spectroscopy (EDS)** analyses were measured using a Thermo Fisher Scientific Apreo 2C scanning electron microscope, equipped with a field emission gun (FEG) and an advanced electron column featuring NICol technology. The microscope is equipped with two Bruker XFlash 60–30 EDS detectors, each with a 30 mm^2^ active detection area, 123 eV energy resolution for Mn Kα, and 45 eV for C Kα. This setup allows for precise point analysis, elemental concentration profiling, and elemental mapping.
- **Solid-state single pulse** ^**27**^ **Al and** ^**29**^ **Si magic angle spinning (MAS) NMR spectroscopy** using a Bruker Avance II 400 MHz Nuclear Magnetic Resonance (NMR) Spectrometer. The analyses were conducted using High-Power Decoupling (HPDEC) for both aluminum (^27^Al) and silicon (^29^Si) nuclei. For the ^27^Al analysis, a resonance frequency of 104.2613 MHz was applied, with a spectral window of 2397.8 ppm centered at 0 ppm. The acquisition consisted of 8192 data points, processed with 16,384 points, using a pulse interval of 5 s and a 90° pulse (p1) duration of 1.250 μs. A total of 1024 scans were performed, with a spinning frequency of 10 kHz. Similarly, for the ^29^Si analysis, the resonance frequency was 79.4866 MHz, with a spectral window of 1258.07 ppm centered at −100 ppm. The acquisition followed the same data point structure but with a longer pulse interval of 60 s and a 90° pulse (p1) duration of 3.500 μs. The analysis also included 1024 scans with a spinning frequency of 10 kHz.

## 3 Results and Discussion

### 3.1 Raw Materials Characterization

XRF, PSD, physisorption analysis, XRD, FT-IR, ^29^Si, and 27NMR, and SEM/EDS characterized the three aluminosilicate sources. The chemical composition determined by XRF and the physical characteristics of these metakaolins are presented in Table 1.

The composition of the aluminosilicate sources has a notable influence on the geopolymerization process.^39–41^ The molar ratio of silicon dioxide (SiO_2_) to aluminum oxide (Al_2_O_3_), directly affects the dissolution in the alkaline solution, depolymerization, condensation of silicate and aluminate monomers, and polycondensation of species-rich in Si–O–Si, Si–O–Al, and Al–O–Al bonds.^12,42,43^ Since the sources of dehydroxylated, kaolin evaluated in this research have significant differences in their chemical specifications, the setting, hardening time, and compressive strength were also affected by the Si/Al molar ratio.

The theoretical formula of dehydroxylated kaolinite (Si_2_O_5_,Al_2_O_2_) provides information about the IV-fold coordination state of the aluminum atom, and the molar ratio of Si/Al is 1.04.^7,12,39^ The MKS source exhibited a Si/Al molar ratio closer to the theoretical value. Otherwise, the MKB powder showed a significant deviation in its Si/Al molar ratio, which directly influenced the amount of alkaline solution and metakaolin used in preparing the geopolymer formulations.^12,42^ As additional information, the specific surface areas of the metakaolins MKS and MKB were 15.64 m^2^/g and 19.19 m^2^/g, respectively (Table 1 and Figure S1). Both aluminosilicates exhibited a Type III isotherm, suggesting weak interactions between the adsorbent and adsorbate.^44^ The median particle size differed moderately, with MKS exhibiting a median of 19.56 μm and MKB measuring 28.90 μm.

The composition of the LiMW was also analyzed by XRF. Table 2 displays the chemical and physical data, and it is clearly possible to see the differences between this byproduct, used as an aggregate source, and the two metakaolins, especially in terms of the content of silicon and aluminum, as well as its notably larger median particle size (141.98 μm) and low specific surface area (0.22 m^2^/g).

Given that geopolymers are primarily noncrystalline, the X-ray patterns of metakaolin can indicate the presence of crystalline phases in the source material. The X-ray diffractograms of the MKS, MKB, and LiMW are shown in Figure 1. The MKS, represented by the green curve, was obtained following the thermal treatment of the Sulfal Kaolin. Thereby, the MKS diffractogram displays a broad diffuse halo, indicating the presence of a disordered or amorphous structure in metakaolinite and confirming that the dehydroxylation of kaolin was essentially achieved.^7,45^

**Figure 1:**
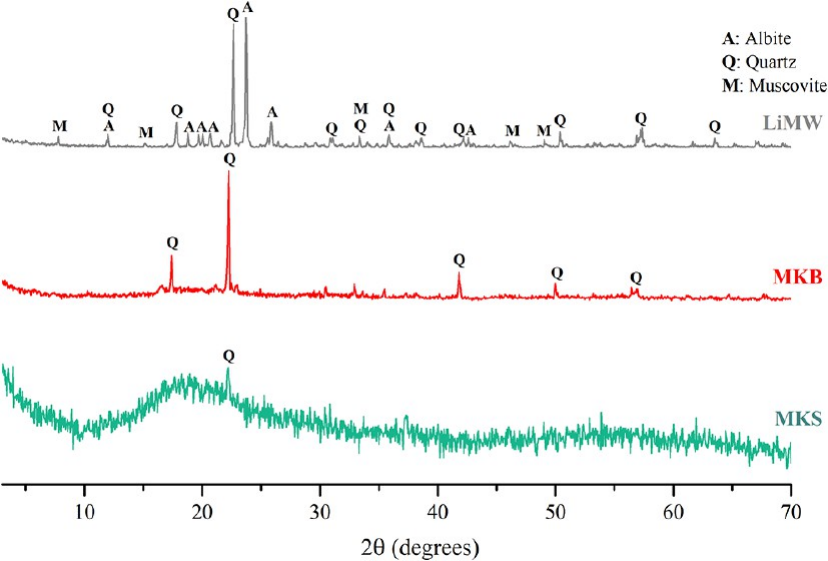
XRD patterns of the MKS, MKB, and LiMW.

As described in Section 2, the kaolin used to produce MKS was thermally activated at 800 °C for 4 h. Although this calcination step is known to be energy-intensive, it was necessary at the early stage of this research to ensure a high degree of amorphization and, consequently, optimal reactivity of the metakaolin. Nevertheless, when compared with clinker production and traditional ceramic processing, the overall synthesis route of geopolymeric materials–including the thermal conversion of kaolin to metakaolin–results in significantly lower carbon emissions, reduced energy consumption, and improved environmental and economic feasibility.^46–48^

The remaining crystallinity percentage in both metakaolins was estimated from XRD data using Origin software, and the approximate value obtained for MKS was 4.9%. In contrast, the commercial metakaolin (MKB), represented by the red curve, had a crystallinity percentage of 25.2%. Although the red diffractogram is primarily composed of indistinguishable peaks, it also exhibits well-defined diffraction peaks associated with the presence of quartz. This significant difference in crystallinity further influences in the geopolymerization reaction, as only approximately 75% of the SiO_2_ measured by XRF in MKB (see Table 1) is available in the amorphous phase.

XRD patterns of LiMW are presented in Figure 1. Mineral composition, estimated by Rietveld’s method, was defined as follows: 57.9% of albite (NaAlSi_3_O_8_), 33.1% of quartz (SiO_2_), and 9.0% of muscovite (KAl_2_(AlSi_3_O_10_)­(F,OH)_2_). This byproduct is generated during the filtration process following the leaching of β-spodumene with sulfuric acid, and during the evaporation step, which removes impurities before the precipitation of Li_2_CO_3_.^3^ The mineralogical composition observed agrees with reported information about spodumene ores,^49,50^ and the chemical composition, as measured by XRF (Table 2), also suggests a high content of SiO_2_, which is directly related to the intensity of the diffraction peaks of albite and quartz.

Infrared spectroscopy was used as a complementary analysis to X-ray diffraction. The infrared spectra and the assignment of the vibrational bands are presented in Figure 2 and Table 3. This technique is useful for understanding how the Si/Al molar ratio in geopolymeric precursors influences the number of tetrahedral aluminum atoms and the frequency of stretching modes.^51,52^ In the case of the MKS, FT-IR confirmed the disappearance of the band at 910 cm^–1^ in kaolinite, related to 6 coordinated Al–OH stretching vibrations, and the replacement by the 4 coordinated Al–O stretching vibrations at 784 cm^–1^.^7,52^ Infrared spectroscopy also proved the dehydroxylation of kaolinite, as indicated by the disappearance of bands between 3700–3600 cm^–1^ and the broadening or undefinition of absorption bands below 1200 cm^–1^, as presented in the MKS spectra.^45^ The MKB spectra does not exhibit significant differences from the MKS spectra. Considering that LiMW is a source of aluminosilicates in geopolymer synthesis, FT-IR spectroscopy revealed the characteristic vibrational bands of aluminosilicates and quartz, particularly in the 800–400 cm^–1^ range, where the most distinguishable vibrations of these components occur (Table 4).

**Figure 2:**
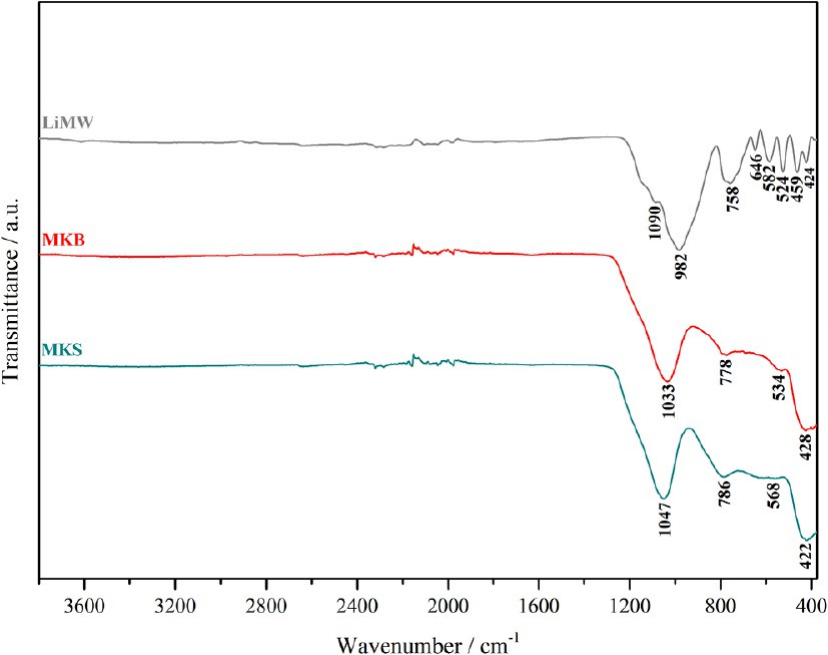
FT-IR spectra of the raw materials.

**Table 4:** Interpretation of Infrared Bands of Aluminosilicate Sources

Wavenumber (cm^–1^)	Assignment	Interpretation	References
3700–3400	ν O–H	Adsorbed water	^45,51,52^
1600–1660	∂ O–H	Adsorbed water	^51–53^
1500–1400	ν_as_ C–O	Carbonate groups	^51–53^
1250–1000	ν_as_ Si–O–Si, Si–O–Al	Aluminosilicates	^21,51,53,54^
1000–970	ν Si–O–K^+^ or Na^+^	Aluminosilicates	^21,52,54,55^
900–920	ν Al–OH	6 coordinated Al	^7,54^
880–840	∂ Si–OH	Aluminosilicates	^52,54^
800–790	ν_sym_ Al–O, ν_sym_ Si–O–Si	4 coordinated Al	^7,54^
700–600	ν_sym_ Si–O–Si, Al–O–Si	Aluminosilicates, quartz	^21,53^
600–400	∂ Si–O–Si, Al–O–Si	Aluminosilicates, quartz	^51,53^

The ^27^Al NMR spectra of the raw materials are shown in Figure 3. The two types of metakaolin, MKS and MKB, exhibit three overlapping resonance signals centered at approximately 58, 28, and 5 ppm, corresponding to tetrahedral, pentacoordinated, and octahedral aluminum environments, respectively.^56–58^ The variations in resonance observed, particularly for the pentacoordinated aluminum units (Al­(V)), may be associated with geometric factors, such as the T–O–T bond angles (where T is Si or Al) in dehydroxylated kaolinite.^59^ Therefore, ^27^Al NMR spectroscopy confirms that the two types of metakaolin contribute differently in terms of Al species during the geopolymer synthesis. The resonance peaks observed around 58 and −3 ppm in the LiMW spectrum are consistent with previous studies and are attributed to the presence of albite and muscovite in the mining waste.^60,61^

**Figure 3:**
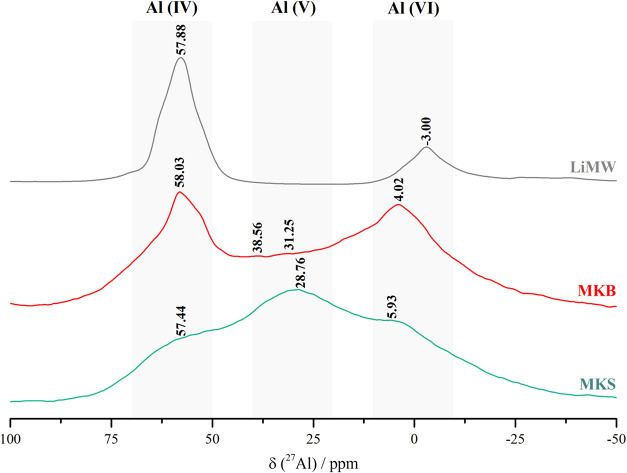
^27^Al NMR spectra of the aluminosilicate precursors: MKS, MKB, and LiMW.

The ^29^Si NMR spectra of the three aluminosilicate precursors, along with their deconvoluted bands using Gaussian–Lorentzian distributions, are presented in Figure 4. The spectra of the metakaolins exhibit a broad and asymmetric resonance centered at approximately −100 ppm, typically attributed to amorphous or pseudoamorphous silicates and aluminosilicates.^57,62^ Metakaolin is primarily composed of Q^4^(1Al) silicon centers,^56,62,63^ whereas spectral deconvolution reveals the presence of other tetrahedral (SiO_4_) units in MKS and MKB, mainly Q^1^, Q^2^, and Q^4^(0Al). As previously discussed, the LiMW used as aggregate in the geopolymer formulations is a heterogeneous and complex material in terms of crystallinity. According to the literature, the moderately broad resonance in the range of −85 to −95 ppm is consistent with ordered feldspars and can be assigned to the presence of albite in the LiMW.^64^ In this same resonance range, earlier studies have attributed the main peak to muscovite.^65^ The deconvoluted band between −100 and −110 ppm is related to the presence of quartz.^57,66^

**Figure 4:**
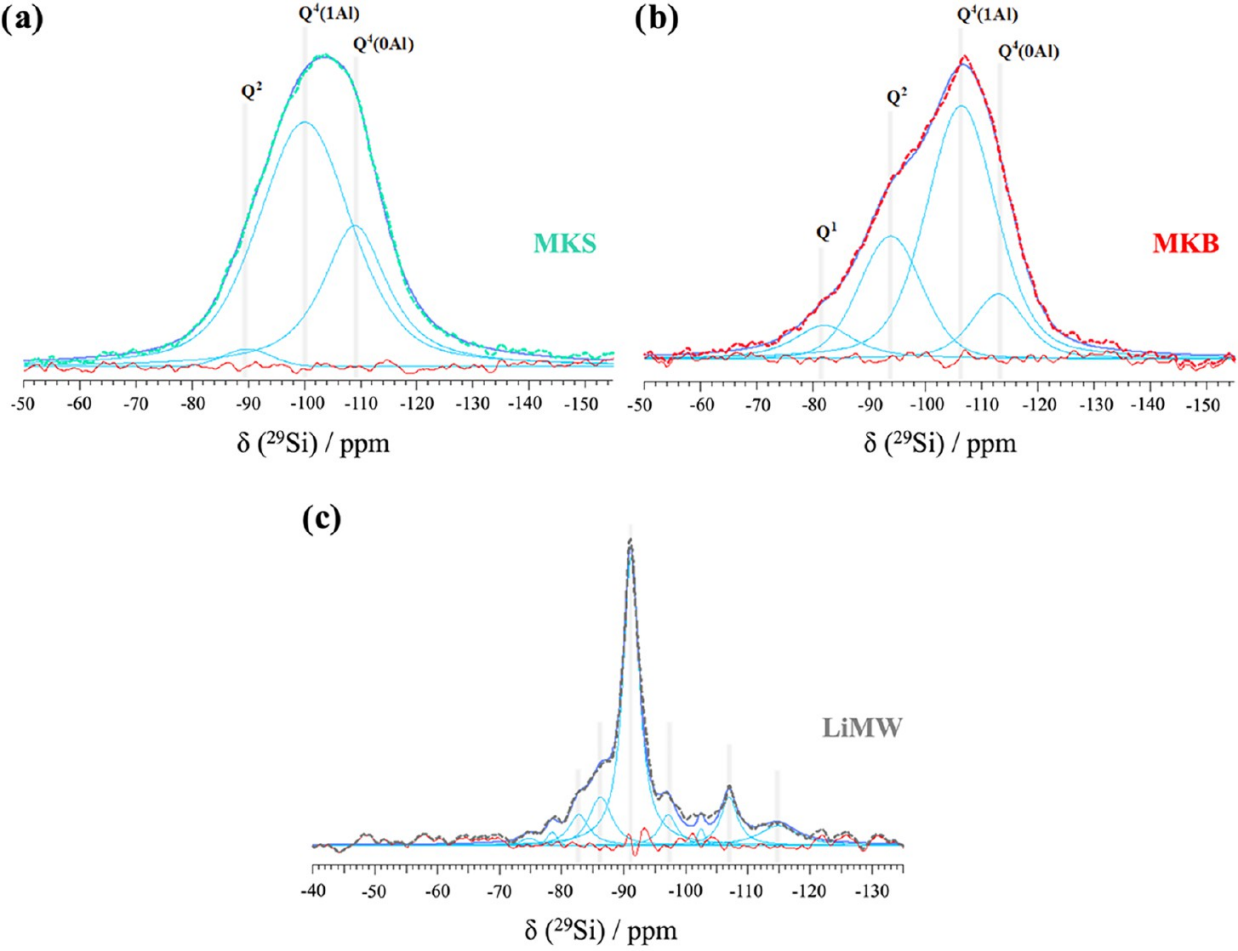
^29^Si NMR deconvoluted spectra of the aluminosilicate precursors (a) MKS, (b) MKB, and (c) LiMW.

As shown in Figure 5, the microstructural analysis of the LiMW revealed a heterogeneous distribution of particles with varying sizes and shapes. EDS analysis (Figure 5c–g) confirmed a homogeneous distribution of Si, Na, and Al in the polished sample, attributed to albite and quartz. The notable distribution of Si agrees with the elevated SiO_2_ content demonstrated by XRF in Table 2. The distinguishable traces of potassium are linked to muscovite, which is present in a smaller proportion in the LiMW compared to albite and quartz.

**Figure 5:**
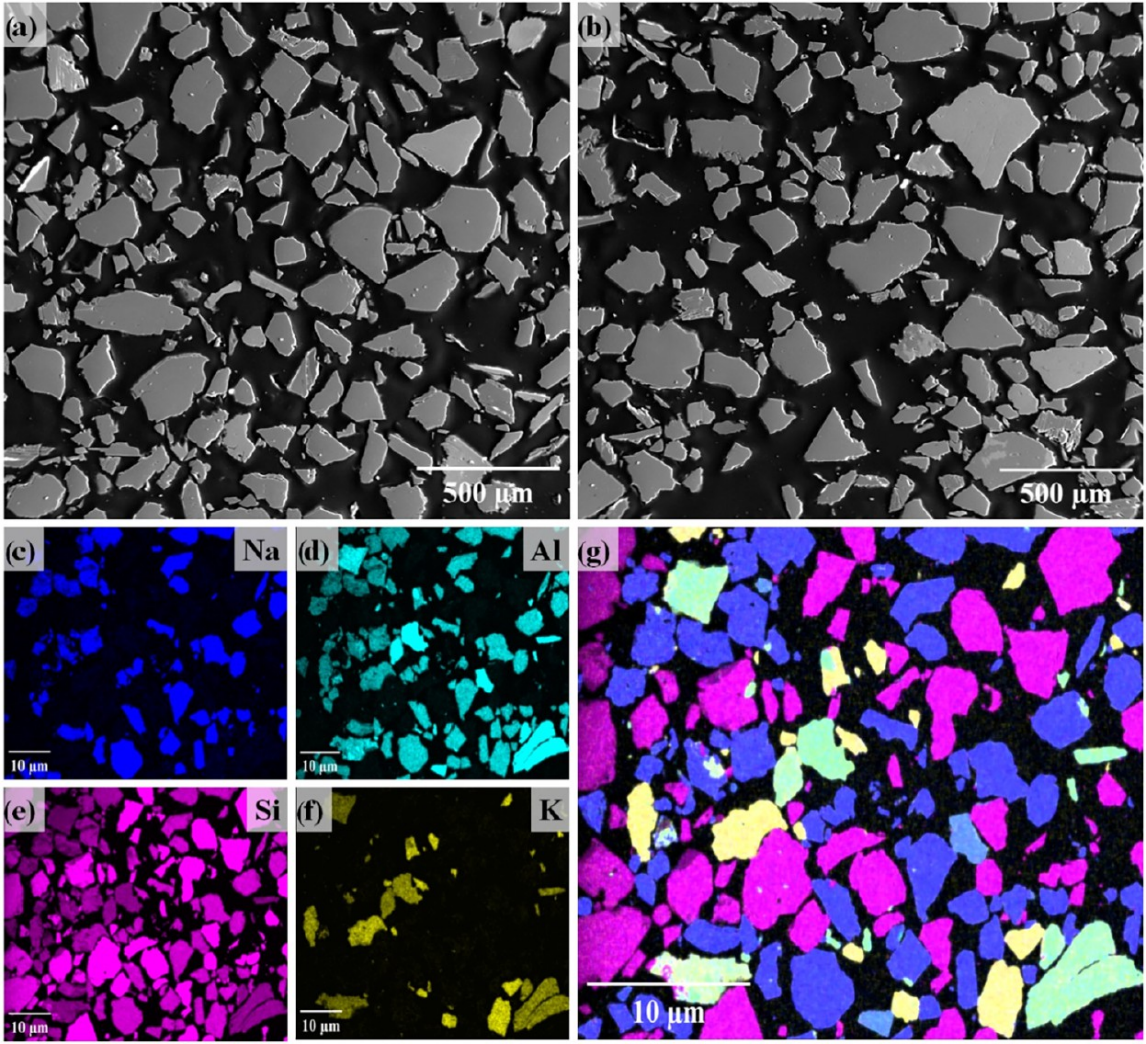
SEM images (a, b) and EDS elemental maps for Na (c), Al (d), Si (e), K (f), and a combined element map (g) of the LiMW.

### 3.2 Influence of Metakaolin and Lithium Mining Waste on Geopolymers’ Compressive Strength and Chemical Properties

#### 3.2.1 Compressive Strength

The physicochemical characteristics of the raw materials, such as mineralogical and chemical composition, degree of crystallinity, molar ratios of SiO_2_/Al_2_O_3_, R_2_O/Al_2_O_3_, and SiO_2_/R_2_O (R refers to Na^+^ or K^+^), the ratio of liquid-to-solid materials, and particle size, directly affect the development of the geopolymer network and its properties.^11,12,35–37^

As mentioned in Section 2.1, a SiO_2_/Na_2_O molar ratio of 1.8 was selected to investigate the influence of LiMW on the compressive strength and chemical properties of geopolymers, incorporating 10, 20, 30, 40, 50, and 60 wt % of this aggregate, and using two types of metakaolin. It is important to emphasize that the amount of metakaolin and alkaline solution was determined based on the chemical composition of the raw materials to establish a Na/Al molar ratio of 1 for all geopolymers. This equilibrium must be respected to obtain a stable geopolymer without free alkali, efflorescence, surface cracking, and blooming^9,20^ (Figure 6). Conversely, achieving the correct balance provides geopolymers with optimal mechanical and chemical properties.

**Figure 6:**
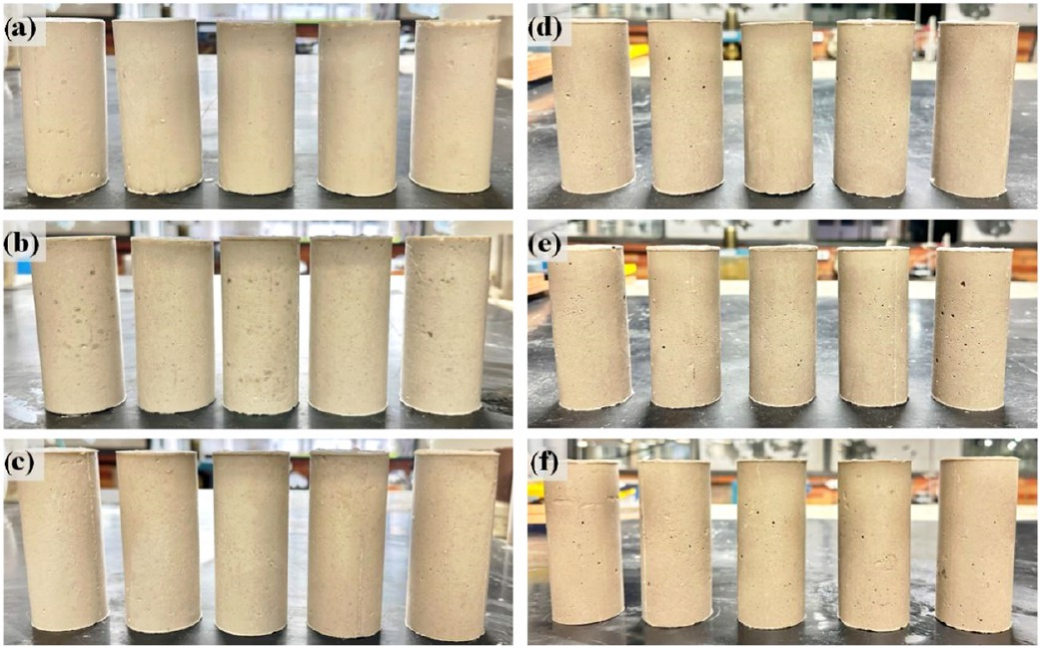
Visual representation of geopolymer samples synthesized under varying conditions. On the left, samples prepared with MKS incorporating (a) 0% LiMW, (b) 40% LiMW, and (c) 60% LiMW. On the right, samples prepared with MKB incorporating (d) 0% LiMW, (e, f) 40%.

In this initial part of the investigation, the samples were submitted to the compressive test after 7 days of curing. Figure 7 presents the results using MKS and MKB as metakaolin precursors, respectively. The maximum compressive strength values were achieved utilizing MKS as a metakaolin precursor, in comparison to the geopolymers synthesized with MKB. If the samples without LiMW were compared, the compressive strength achieved was 45.4 and 23.7 MPa for the geopolymers produced with MKS and MKB, respectively.

**Figure 7:**
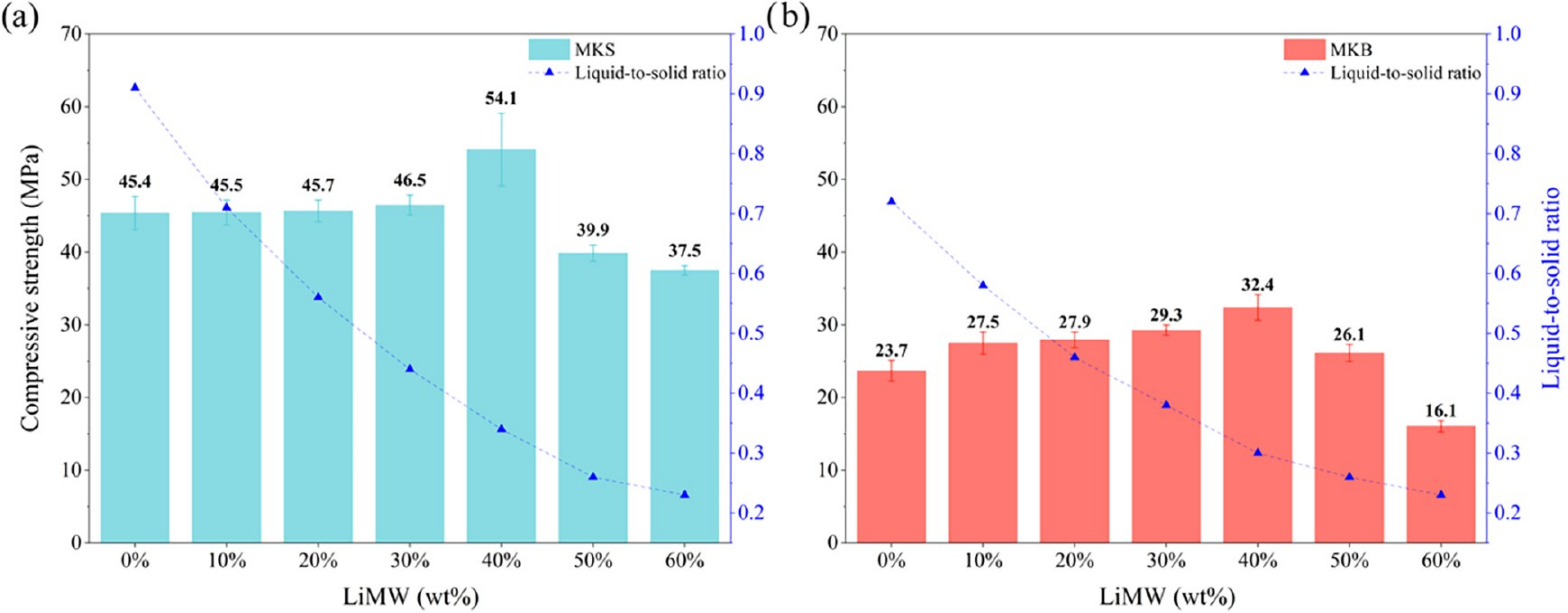
Compressive strength of the geopolymers synthesized with (a) MKS and (b) MKB at varying LiMW proportions.

Several studies have been conducted in the geopolymer field, and it is well established that the properties of geopolymeric binders, including microstructure and mechanical performance, are highly dependent on factors such as the raw material composition, the availability of alumina and silica from the aluminosilicate source, the Si/Al molar ratio, the type of alkaline reactants and its content, the curing time and temperature.^11,39,55,67–74^ Autef et al.^75^ reported that the presence of amorphous versus crystalline silica (such as quartz) influences both the geopolymerization reaction and the resulting mechanical properties. He et al.^76^ also reported the effect of varying the Si/Al ratio from 2.0 to 4.0 on the microstructure and mechanical properties–flexural strength and Young’s modulus–of metakaolin-based geopolymers. Considering these findings, the compressive strength results reported here are consistent with previous studies.

Although the MKB has a higher amount of SiO_2_, its source of silica is not completely amorphous and reduces its reactivity during the geopolymerization, compared to the more amorphous silica present in the MKS. As a result, the nanostructure and engineering properties of the developed geopolymer are adversely affected. Amorphous aluminosilicate structures in raw materials are desirable for achieving long-term properties and optimal mechanical strength values, since the depolymerization of silicate and aluminate species in the alkaline solution proceeds to a significant extent.^11,20,55^

Increasing the LiMW proportions in metakaolin-based geopolymers synthesized with MKS or MKB, the molar ratio of Si/Al is increased by the replacement of metakaolin with LiMW, which has an elevated amount of crystalline SiO_2_ (See Tables 1 and 2). Due to its mineral and chemical composition, the increase in LiMW proportion in geopolymer formulations is related to higher crystallinity, lower silica and aluminum reactivity, and larger median particle size. According to the literature, geopolymers binders synthesized with highly crystalline precursors tend to form undesirable and unreacted species, caused by their slow kinetic dissolution rate in alkaline media, leading to a delayed strength development and poor matrix integrity.^11,12,20^

However, the addition of LiMW does not have the preferred characteristics of aluminosilicate precursors; the incorporation of mining waste, up to an adequate proportion, significantly enhanced the compressive strength of both syntheses. Given the nature of the aggregate, especially regarding the unavailable amorphous silicon and aluminum oxides, this improvement in mechanical properties of the resulting geopolymers may be attributed to the crystal particle packing theory (filler effect), which develops the formation of a denser microstructure.^77,78^ The best compressive strength was achieved by incorporating 40% of mining waste in both syntheses. The geopolymer produced with MKS obtained 54.1 MPa, up to 1.6 times higher than that prepared with MKB. It must be mentioned that these LiMW incorporations did not hinder the workability or homogeneity of the geopolymer mixture, even with the decrease in the liquid-to-solid ratio. The proportion of alkaline solution was rebalanced to all formulations to maintain the optimal molar ratio of Na_2_O/Al_2_O_3_ and SiO_2_/Na_2_O.

As disclosed, the geopolymers exhibiting high compressive strength values were evaluated after 14 and 28 days of curing. Figure 8. represents the influence of curing time under ambient temperature for the geopolymers synthesized with 40% of LiMW. The compressive strength developed at 28 days was 59.3 and 43.7 MPa for the geopolymers prepared with MKS and MKB, respectively. Some studies using various sources of lithium mining waste and different curing conditions have also reported an improvement in compressive strength over the curing period.^29,30^ Furthermore, the more evident enhancement of engineering properties in the MKB geopolymer can be attributed to the retarded setting time of the mixture and the formation of stronger structure, resulting from the physicochemical characteristics of the metakaolin used.

**Figure 8:**
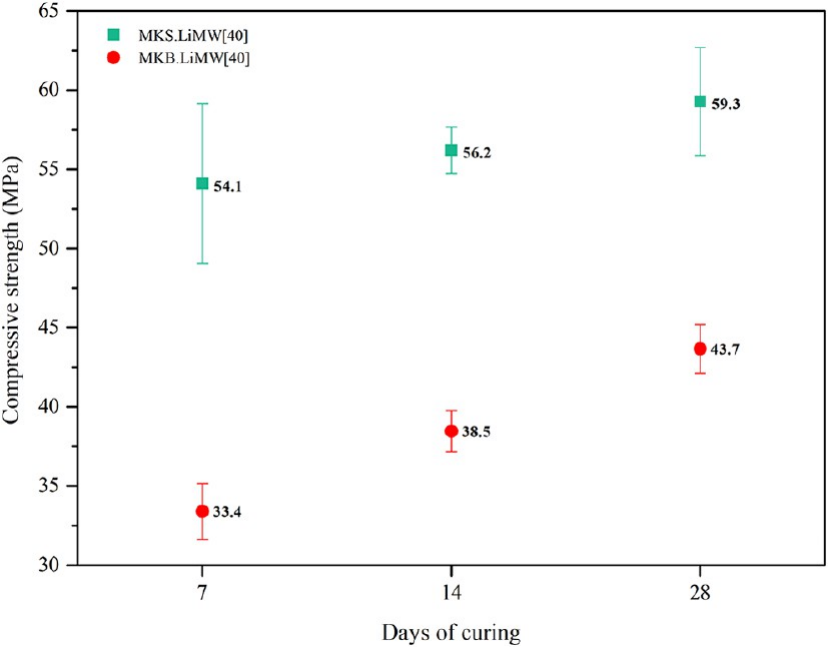
Effect of curing time on compressive strength at 7, 14, and 28 days.

Three explanations can be hypothesized for the enhancement of engineering properties in the geopolymers synthesized with LiMW, and the notable difference in results when comparing the two types of metakaolin used in this research. The aluminate component sourced from aluminosilicates (such as metakaolin, fly ash, blast furnace slag), under alkaline conditions, readily promotes the formation of [Al­(OH)_4_]^−^ tetrahedra species and aluminosilicate network structures during the initial stages of geopolymerization. These aluminosilicate species have a superior electrostatic interaction with charged hydroxyl groups than silica, so the kinetics of the condensation reaction will occur more efficiently.^10,20,79^

Given the elevated amorphous aluminate content in MKS, the hydrolysis rate of Al is possibly occurring as expected, even with the decrease in metakaolin and alkaline solution content, simultaneously with the LiMW proportion increase. Meanwhile, silica release is also a determinant in geopolymer chemistry. The rate of silica availability is responsible for leading to faster depolymerization and control of the behavior of solid aluminosilicate to geopolymeric binder conversion.^40,80^ Fernández-Jiménez et al.^68^ and Duxson et al.^56^ reported that higher silica participation in the geopolymer reaction begins when an elevated amount of alkaline solution is available in the synthesis. The dissolution rates of alumina and silica are interdependent, although not necessarily congruent. Accordingly, the local speciation of dissolved Al and Si in alkaline solution strongly depends on the nature of the solid aluminosilicate source and the identity of the silicate reactant.

Even though MKS has a lower silica content than MKB, its silica is amorphous. When this characteristic is combined with the silica available in the alkaline solution and the packing effect induced by the incorporation of LiMW, the mechanical strength is enhanced. This chemical fact also explains the distinguishable divergences in the final mechanical properties observed when the metakaolin powder was varied while maintaining the same amount of LiMW.

As shown in Figure 7, the improvement in mechanical characteristics with increasing LiMW incorporation (from 0 to 40%) is accompanied by a decrease in the water-to-solid ratio in both syntheses. In geopolymeric materials, excess water is problematic as it remains outside the three-dimensional geopolymer network, acting as a lubricant element that only enhances the mixture’s workability.^7,80^ Nevertheless, this unbound water also negatively affects the density and porosity, while facilitating faster evaporation and the potential formation of surface cracks.^79,81^ The optimal liquid-to-solid ratios reached using MKS and MKB were 0.34 and 0.30, respectively.

Several researchers have reported the use of different mining wastes as aggregates in geopolymerization reactions. Table 5 presents synthesis data and the corresponding compressive strengths for these selected waste sources, demonstrating that the mechanical performance achieved in this study is comparable to or better than those reported previously.

**Table 5:** Geopolymers Synthesized from Different Waste Sources under Various Synthesis Conditions

Waste source	Waste proportion (wt %)	Alkaline reactants	Curing conditions	Compressive strength	References
Tungsten	90%	NaOH, Na_2_SiO_3_, and Ca(OH)_2_	Room temperature	25–40 MPa (after 7–28 days of curing)	^82^
Tungsten + Blast furnace slag	10–90%	NaOH, Na_2_SiO_3_, KOH, and Ca(OH)_2_	Initially cured at 60 °C for 24 h, then at room temperature	15–30 MPa (after 7–28 days of curing)	^31^
Fly ash + Quartz	70–80%	NaOH and Ca(OH)_2_	Room temperature	10–30 MPa (after 7–28 days of curing)	^83^
Lithium slag + Fine-grained lead–zinc tailings	64%	NaOH	Initially cured for 12 h at 25–100 °C, then at room temperature	3–52 MPa (after 7–28 days of curing)	^30^
Lithium slag + Blast furnace slag + Fly ash + Kaolin	25–100%	NaOH and Na_2_SiO_3_	Room temperature	0–84 MPa (after 7–28 days of curing)	^29^
Iron ore tailings	50–60%	NaOH and Na_2_SiO_3_	Room temperature	24–38 MPa (after 28 days of curing)	^27^
Iron ore tailings	50%	NaOH and Na_2_SiO_3_	Room temperature	59–65 MPa (after 7 days of curing)	^15^

#### 3.2.2 X-ray Diffraction Analysis

The diffraction patterns obtained for geopolymers synthesized with LiMW and the two types of metakaolin are presented in Figures 9 and 10.

**Figure 9:**
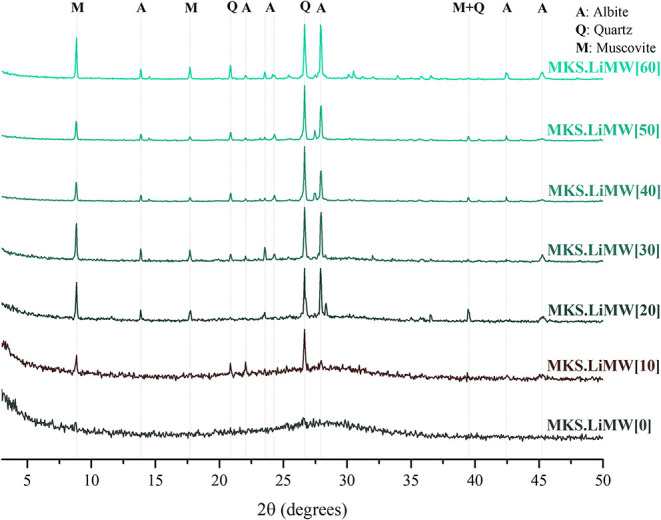
XRD patterns of the geopolymers synthesized with MKS at varying LiMW proportions.

**Figure 10:**
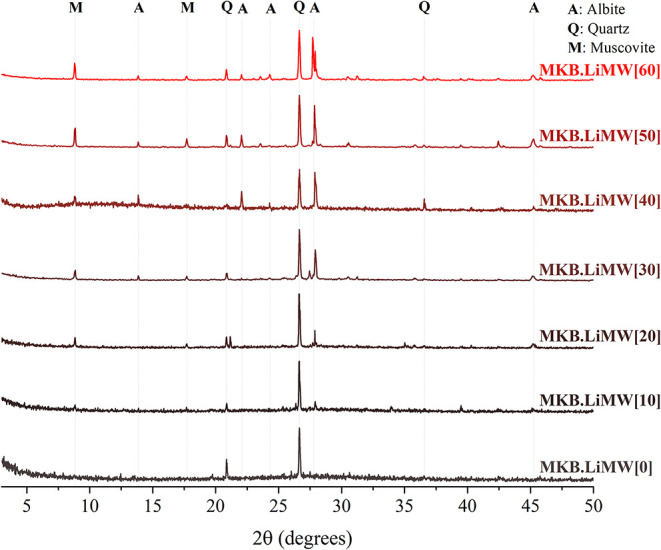
XRD patters of the geopolymers synthesized with MKB at varying LiMW proportions.

Analysis of the presented XRD data highlights significant distinctions between the geopolymers synthesized using only metakaolin and those incorporating LiMW. The initial diffractogram of geopolymer produced from MKS without LiMW incorporation has a broadened diffuse halo characteristic of amorphous geopolymers.^7,79,84^ The geopolymer synthesized from MKB also has an amorphous diffractogram, but there is a marked presence of quartz related to the chemical composition of this precursor. As the LiMW proportion increases, the intensity of well-defined diffraction peaks associated with quartz, albite, and muscovite also increases. Since these crystalline phases are present in the mining waste, the results are coherent with previous findings regarding the use of nonamorphous aluminosilicate sources.^78^

Considering that no process was applied to LiMW to enhance its reactivity, such as mechanical and chemical activation, thermal treatment, or separation,^12^ these diffraction patterns align with the reduction in metakaolin content, the primary amorphous contributor to the geopolymeric reaction, followed by adding LiMW, a crystalline aluminosilicate, as an aggregate (see Table 3). Regarding crystallographic information, no substantial contrasts are observed among the diffractograms of geopolymers synthesized with MKS or MKB, using 10 to 60% of LiMW. In this investigation, XRD analyses did not provide a detailed chemical justification for the positive behavior of LiMW incorporation on geopolymers’ compressive strength.

#### 3.2.3 FT-IR Spectroscopy Analysis

FT-IR spectroscopy is a valuable tool for assessing silico-aluminate bonds and the framework structures formed in the geopolymers. Figures 11 and 12 present the FT-IR spectra obtained for geopolymers synthesized in both syntheses. Analysis of the presented XRD data highlights significant distinctions between the geopolymers synthesized using only metakaolin and those incorporating LiMW. The initial diffractogram of geopolymer produced from MKS without LiMW incorporation has a broadened diffuse halo characteristic of amorphous geopolymers.^7,79,84^ The geopolymer synthesized from MKB also has an amorphous diffractogram, but there is a marked presence of quartz related to the chemical composition of this precursor. As the LiMW proportion increases, the intensity of well-defined diffraction peaks associated with quartz, albite, and muscovite also increases. Since these crystalline phases are present in the mining waste, the results are coherent with previous findings regarding the use of nonamorphous aluminosilicate sources.^78^

**Figure 11:**
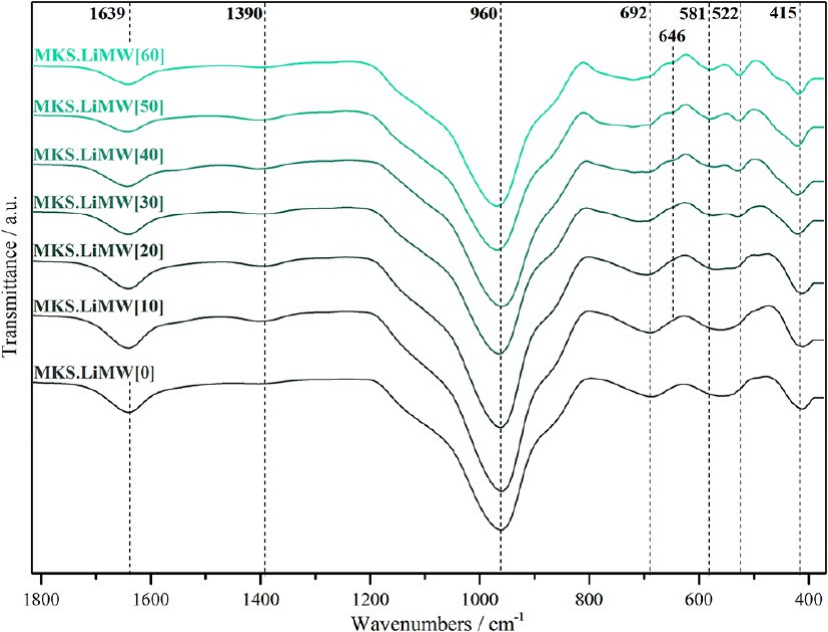
FT-IR spectra of the geopolymers synthesized with MKS at varying LiMW proportions.

**Figure 12:**
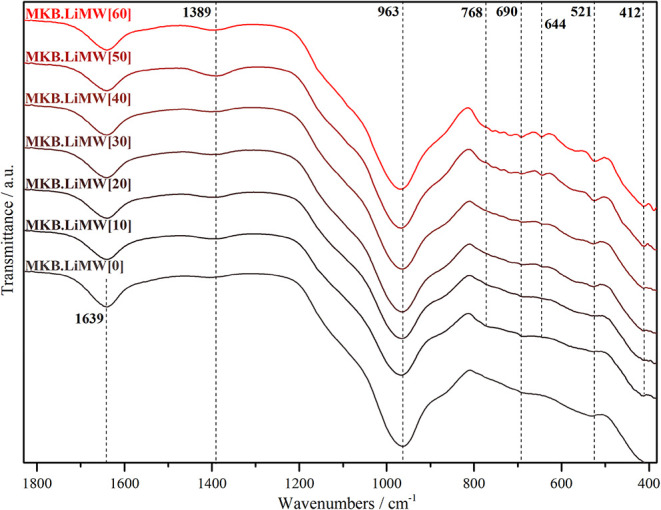
FT-IR spectra of the geopolymers synthesized with MKB at varying LiMW proportions.

In all spectra, using MKS or MKB, several transmission bands do not differ significantly, and the vibrational bands can be identified in a similar range of wavenumbers. Bands in the 1660–1600 cm^–1^ region are related to bending vibrations of a hydroxyl group.^21,51,52^ Bands in the 1500–1400 cm^–1^ region are associated with CO_3_ groups.^51,52,85^ It is important to emphasize that the spectrum of all geopolymers synthesized showed a carbonate group band, even if it was observed with lower peak intensity. XRD analysis did not recognize this carbonation phase. The efflorescence phenomenon occurs through a free alkali reaction with *HCO*_3_^–^ or *CO*_3_^2–^, to form alkali carbonate phases.^9,86^ This carbonation process affects the structural integrity of the geopolymer network, leading to a decrease in mechanical properties.^29,51,86^

The most relevant wavenumbers associated with geopolymerization are based on the 1200–400 cm^–1^ spectral range. The spectra of MKS and MKB show the typical vibrations for all silico-aluminate structures. Bands in the 1200–900, 800–600, and 600–400 cm^–1^ regions are assigned to the asymmetric, symmetric, and bending vibrations of Si–O–Si (Al) linkages, respectively.^21,51,52,54^ In all spectra, the most intense band, 970–960 cm^–1^, corresponds to internal vibrations of Si–O–Si and Si–O–Al of the geopolymeric materials.^14,52,55^ Additionally, in the spectra, the main band in the region of 1100 cm^–1^, associated with metakaolinite, disappeared, confirming that the geopolymerization process was successfully carried out.^45,51,52^

Some geopolymer spectra synthesized with high LiMW proportion exhibited small shifts in peak position at about 960 cm^–1^, which is related to the silica availability in the starting material and subsequent formation of Al-rich and Si-rich gel.^21,36^ Bands at approximately 650, 580, and 524 cm^–1^ are more pronounced in the syntheses with a high LiMW content (50–60%). These bands correspond to the symmetric and bending vibrations of Si–O–Si (Al) bonds related to the presence of aluminosilicates and quartz in the mining waste.^46,47^

Overall, FT-IR spectroscopy confirmed the formation of geopolymeric materials in both syntheses. The incorporation of LiMW did not affect the geopolymerization; even so, as the proportion of the aggregate increased in the syntheses, the appearance of unreacted compounds from this byproduct became more evident in all infrared spectra, as previously observed in the XRD analysis.

#### 3.2.4 ^27^Al and ^29^Si NMR Spectroscopy Analysis

To investigate the chemical structure of the ^27^Al and ^29^Si species, the geopolymers with the best compressive strength, formulated with LiMW, using MKS and MKB as the metakaolin precursor, and the materials without aggregate, were chosen. The ^27^Al NMR spectra of the four selected geopolymers are shown in Figure 13.

**Figure 13:**
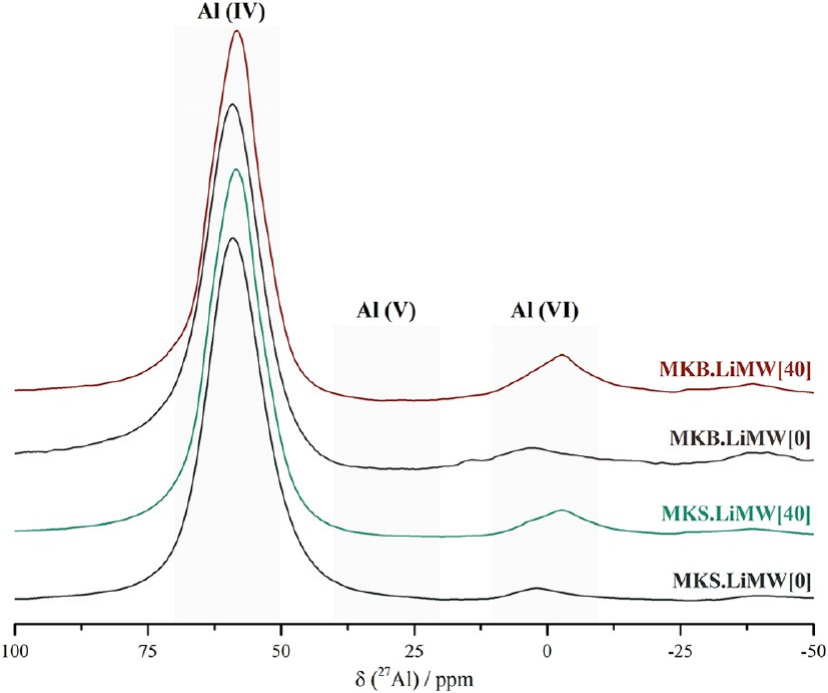
^27^Al NMR spectra of the geopolymers synthesized with MKS and MKB, with and without incorporating 40% of LiMW.

According to previous studies, the predominant resonance signal at approximately 60 ppm in metakaolin-based geopolymers corresponds to the tetrahedrally coordinated aluminum (Al­(IV)), indicating the formation of a 3D silico-aluminate framework.^57,63,66^ Resonance signals observed in the range of 40 to −10 ppm are assigned to unreacted metakaolin species, including penta (Al­(V)) and octahedrally (Al­(VI)) coordinated aluminum.^57,66^ In the spectrum of geopolymers synthesized with LiMW, the broader signal centered at −3 ppm, characterized by its width and full width at half-maximum (FWHM), matches the same resonance observed in the ^27^Al NMR spectrum of the aggregate LiMW (see Figure 3). This indicates that the Al­(VI) units originally present in LiMW remained unreacted during the geopolymerization process. Despite the consistent spectral features of LiMW, using MKS or MKB as a metakaolin precursor, no substantial differences are identified between the materials synthesized without LiMW, demonstrating that the geopolymeric binders were successfully formed in both syntheses.

The ^29^Si NMR spectra of the four selected geopolymers, along with their deconvoluted bands using Gaussian–Lorentzian distributions, are presented in Figures 14 and 15. All the spectra exhibit a broad and asymmetric resonance characteristic of amorphous or pseudoamorphous structures.^57,87^ The different molecular arrangements and degree of condensation of the tetrahedral silicon species are associated with the raw materials used in the geopolymer mixture, and are described by Q*^n^* (*m*Al) units, where “n” is polymerization degree of the Si tetrahedra, and “*m*” is the number of adjacent Al atoms connected to a SiO_4_ unit (0 ≤ *n* ≤ *m* ≤ 4).^21,51,57^

**Figure 14:**
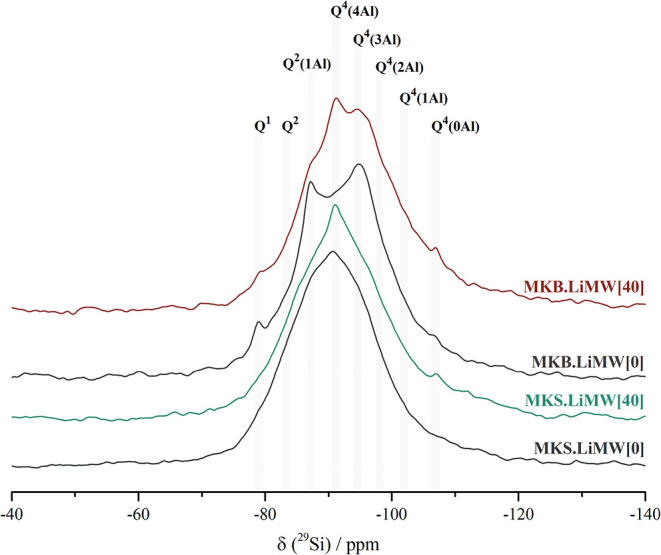
^29^Si NMR spectra of the geopolymers synthesized with MKS and MKB, with and without incorporating 40% of LiMW.

**Figure 15:**
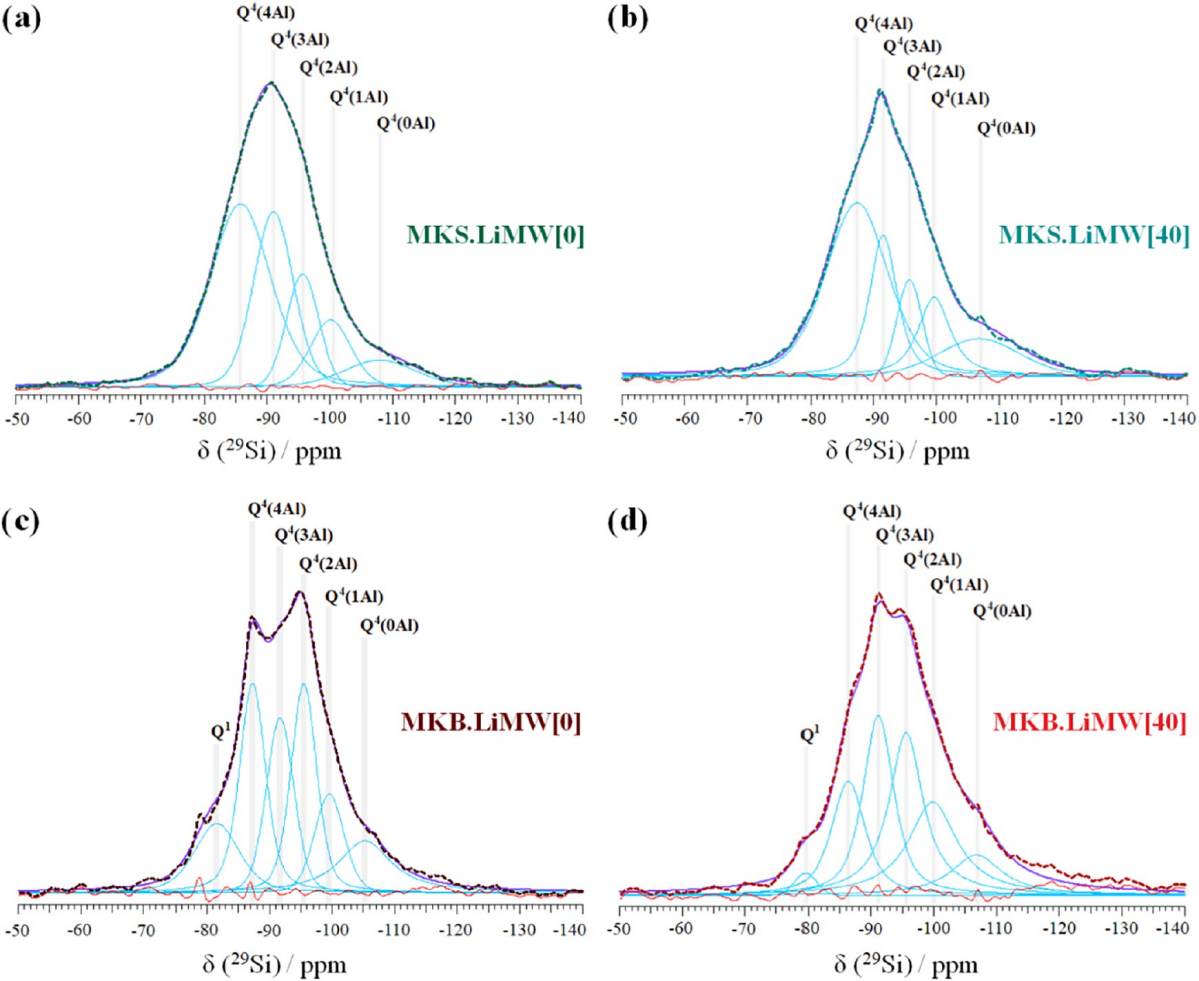
^29^Si NMR deconvoluted spectra of the geopolymers synthesized with MKS and MKB, with and without LiMW. (a) MKS.LiMW[0], (b) MKS.LiMW[40], (c) MKB.LiMW[0], and (d) MKB.LiMW[40].

According to reported studies, silicate frameworks are distinguished by their chemical shifts: Q^4^(4Al) [resonates between −85 and −89 ppm], Q^4^(3Al) [resonates between −89 and −94 ppm], Q^4^(2Al) [resonates between −94 and −99 ppm], Q^4^(1Al) [resonates between −99 and −105 ppm], and Q^4^(0Al) [resonates between −105 and −120 ppm].^21,57,58,87^ In addition, silicon units with a lower degree of condensation are denoted by Q*^n^*, such as Q^1^ and Q^2^, and resonate at higher chemical shift values, typically in the range of −73 to −85 ppm.^51,57,66,88^ In both syntheses, the higher chemical shift value of the main ^29^Si resonance peak and the low intensity at −100 ppm observed in all synthesized geopolymers suggests a low presence of Q^4^(1Al) units, which may result from the almost complete consumption of the metakaolin during the geopolymerization process, and the replacement of Si by Al within the first coordination sphere of central Si atoms.^62,63^ Otherwise, the peak observed at −107 ppm in the spectra of geopolymers synthesized with LiMW, using either MKS or MKB as the aluminosilicate precursor, is attributed to unreacted quartz present in the metakaolin and the mining waste aggregate. Only the geopolymer produced with MKS without LiMW does not show this peak. These results are consistent with the XRD data obtained (see Figures 9 and 10), which confirmed that increasing the LiMW proportion in the geopolymer mixture leads to higher crystallinity and the presence of unreacted components, such as quartz, albite, and muscovite.

Table 6 summarizes the assignments of the Q^4^(*m*Al) species identified in the spectra, along with their estimated quantitative proportions. As can be seen in Figure 15, all deconvoluted spectra are predominantly composed of highly polymerized Q^4^ units. Only a minor contribution from low-condensed Q^1^ species, at approximately −80 ppm, is observed in the geopolymers synthesized with MKB. The presence of these Q^1^ species is commonly attributed to residual unreacted metakaolin or to terminal silanol (Si–OH) groups.^51^

**Table 6:** Quantification of the Q*^n^* (*m*Al) Species Obtained by Deconvoluting the ^29^Si NMR Spectra of the Four Selected Geopolymers

Geopolymers		Q^4^(4Al)	Q^4^(3Al)	Q^4^(2Al)	Q^4^(1Al)	Q^4^(0Al)	Q^1^
MKS.LiMW[0]	δ (ppm) %	–85.73	–90.98	–95.65	–100.10	–107.91	-
		43.58	25.95	12.84	10.15	7.47	
MKS.LiMW[40]		–87.33	–91.54	–95.71	–99.66	–106.82	-
		46.79	18.58	9.27	12.89	12.47	
MKB.LiMW[0]		–87.29	–91.70	–95.49	–99.55	–105.26	–81.59
		22.62	18.38	21.53	11.14	13.53	12.81
MKB.LiMW[40]		–86.37	–91.15	–95.56	–99.80	–106.78	–79.61
		16.51	23.76	26.36	21.82	9.92	1.63

Quantitative comparison further indicates a notably higher fraction of Q^4^(4Al) units in the geopolymers synthesized with MKS. These Al-rich, cross-linked gel structures, combined with the reduced less polymerized components, likely contribute to the observed differences in the engineering properties of the geopolymers produced in this study.

As discussed previously, the availability of amorphous silicon and aluminum oxides–sourced from aluminosilicates and alkaline solution–is determinant in the formation of three-dimensional aluminosilicate network structures in geopolymeric systems. The dissolution and hydrolysis of aluminum species under a high number of soluble silicates in the geopolymer mixture is associated with faster depolymerization kinetics and conversion of the aluminosilicate precursors into geopolymer gel.^36,70^ This process facilitates the formation of a highly polymerized tectosilicate framework characterized by Si–O–Si (Al) linkages.^88,89^

Considering that the geopolymers were synthesized under varying compositional conditions, the spectral deconvolution and quantitative ^29^Si NMR analysis, provide insight into the structural origin of the observed mechanical performance. The superior compressive strength exhibited by the MKS-based geopolymers can be attributed to a higher degree of silicate polymerization and more homogeneous distribution of Q^4^(*m*Al) units, enhancing connectivity and reducing structural defects, consequently, the resulting matrix is denser and more mechanical robust. Furthermore, consistent with the FT-IR findings, the ^29^Si spectral data demonstrate that the incorporation of 40% of LiMW does not compromise the integrity of the aluminosilicate’s framework.

#### 3.2.5 Scanning Electron Microscopy and Energy Dispersive Spectroscopy

Microchemistry and microstructure investigations were additionally performed on the samples with the highest compressive strength and on those synthesized without incorporating LiMW. The SEM images and EDS mapping (Figures 16–19) correspond to the innermost part of the crushed geopolymers obtained after the rupture tests, post 7 days of curing. The resembling images exhibit a dense morphology with some small particles dispersed on the surface.

**Figure 16:**
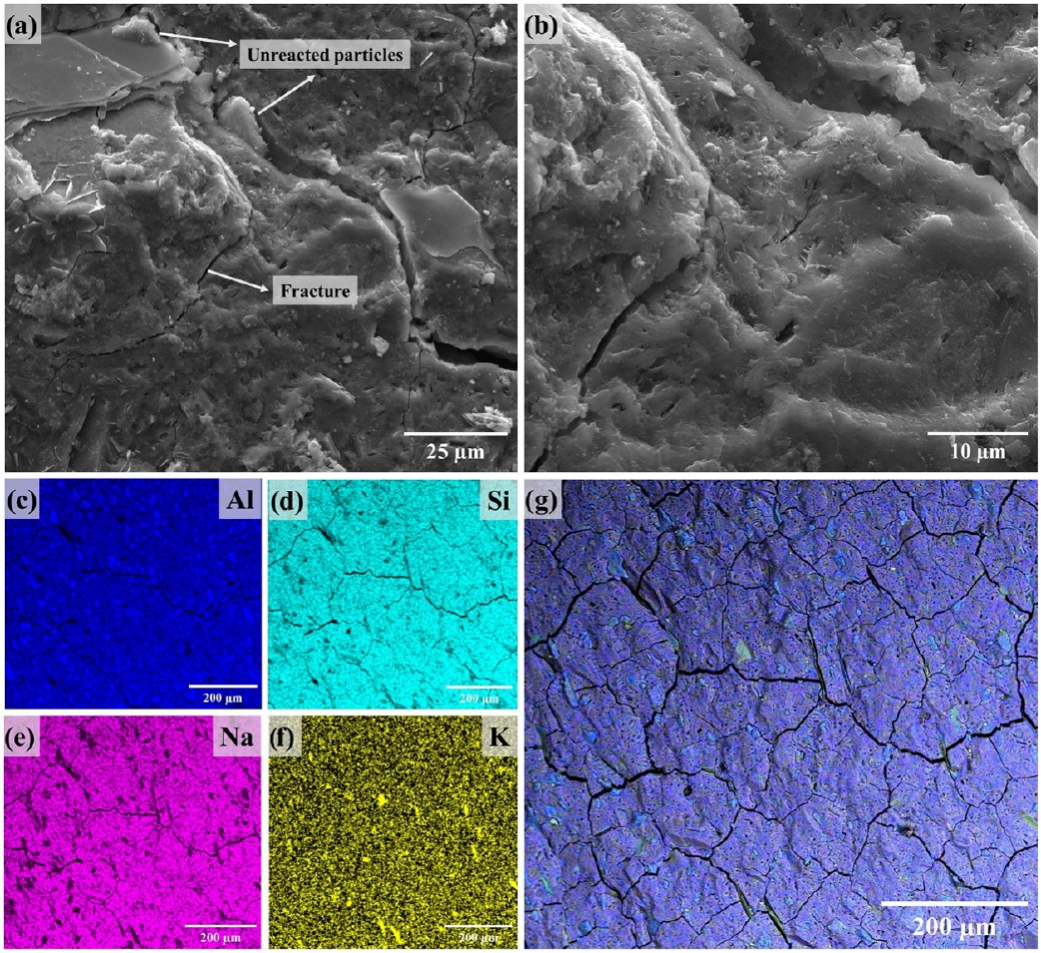
SEM images (a, b) and EDS (c–g) mapping of the geopolymer MKS.LiMW[0].

According to Figures 16 and 17, when the geopolymer is synthesized with MKS powder, the internal area has a lesser presence of these granular particles. This microstructural aspect is directly determined by the hydrolysis of Al and Si and the formation of geopolymer gel during the synthesis.^19,84,90,91^

**Figure 17:**
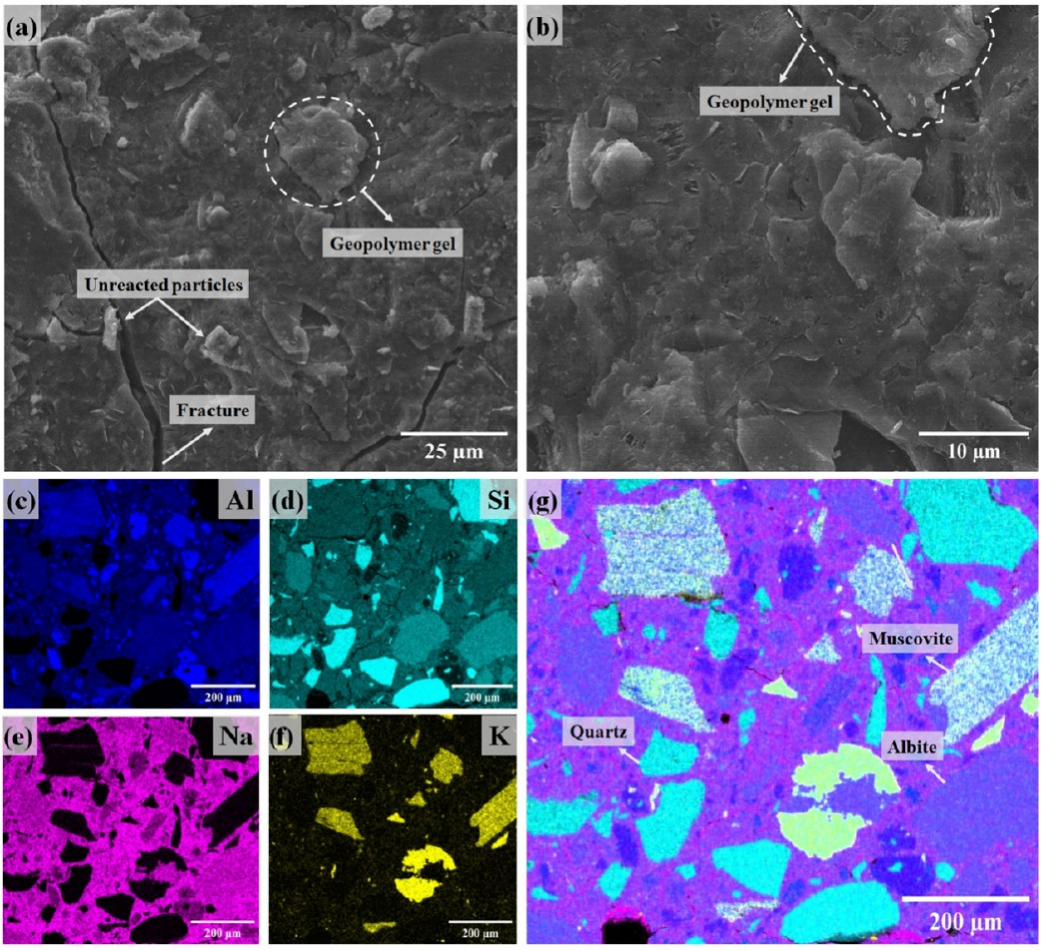
SEM images (a, b) and EDS mapping (c–g) of the geopolymer MKS.LiMW[40].

Given that the materials produced with 40% of mining waste demonstrated the best mechanical performance, it is proposed that the unreacted LiMW particles, due to their crystalline structure and large particle size, contribute to the arrangement and filling of voids within the geopolymer structure. Simultaneously, the possible gaps formed from these large particles of the aggregate appear to be surrounded by the geopolymer gel, contributing to a more solid microstructure. Researchers using different quartz-rich aggregate sources, including marble and basalt powder, have also observed the strengthening in the geopolymer structure caused by the packing effect.^78,92,93^

The disrupted interaction between the coarse and small particles from the Al-rich and Si-rich gel in the matrix arrangement is known as the wedging effect.^77^ This occurs when the large particles are dominant in the system.^77^ Therefore, the physicochemical characteristics of the metakaolin used in these formulations appear to be more capable of forming stable linkages between the LiMW compounds and the metakaolin-based geopolymer, thereby reducing pore formation and resulting in a material with long-term properties.

A homogeneous distribution of Al, Si, Na, and K is demonstrated by EDS analysis (Figure 16c–g) of the MKS-based geopolymer without LiMW. After incorporating the aggregate, EDS mapping (Figure 17c–g) clearly reveals the distinction between areas predominantly composed of geopolymer gel, characterized by the homogeneous distribution of Al, Si, Na, and K, and areas with elevated concentrations of Al, Si, and K, which are associated with the unreacted presence of albite, quartz, and muscovite. Regarding the FT-IR spectroscopy, although the spectrum of this sample exhibited a band associated with the carbonate groups, EDS analysis does not indicate a higher amount of Na in specific areas and subflorescence.^94^

The molecular structure of the aluminosilicate precursors is decisive in the reactivity of the minerals involved in the alkaline geopolymer reaction. Considering the distinct physicochemical properties of the MKB, Figures 18 and 19. display the microstructure and elemental distribution of the geopolymers produced with this metakaolin. The SEM images of the sample synthesized incorporating LiMW have a denser morphology, which is similar to the images obtained for the geopolymer synthesized with MKS and 40% of mining waste. Even though it is important to emphasize the more noticeable presence of unreacted and granular particles distributed on the surface of both specimens.

**Figure 18:**
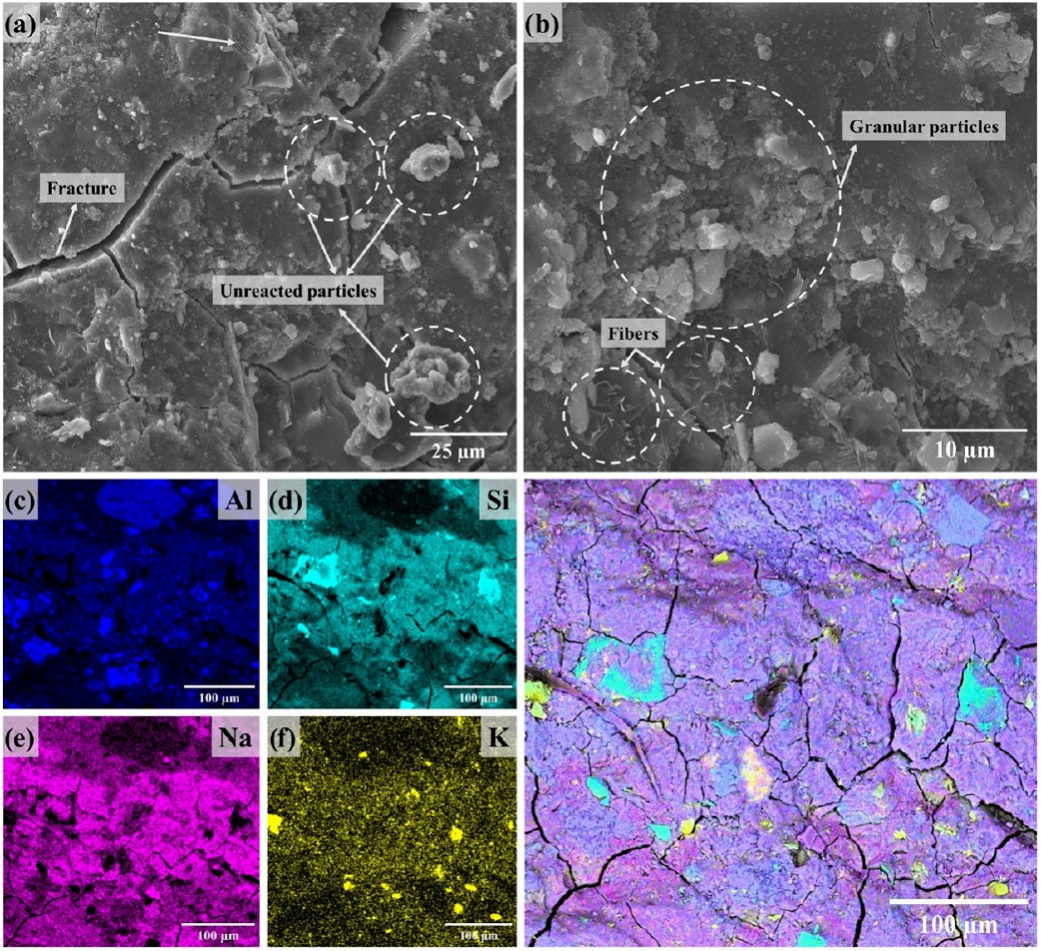
SEM images (a, b) and EDS mapping (c–g) of the geopolymer MKB.LiMW[0].

**Figure 19:**
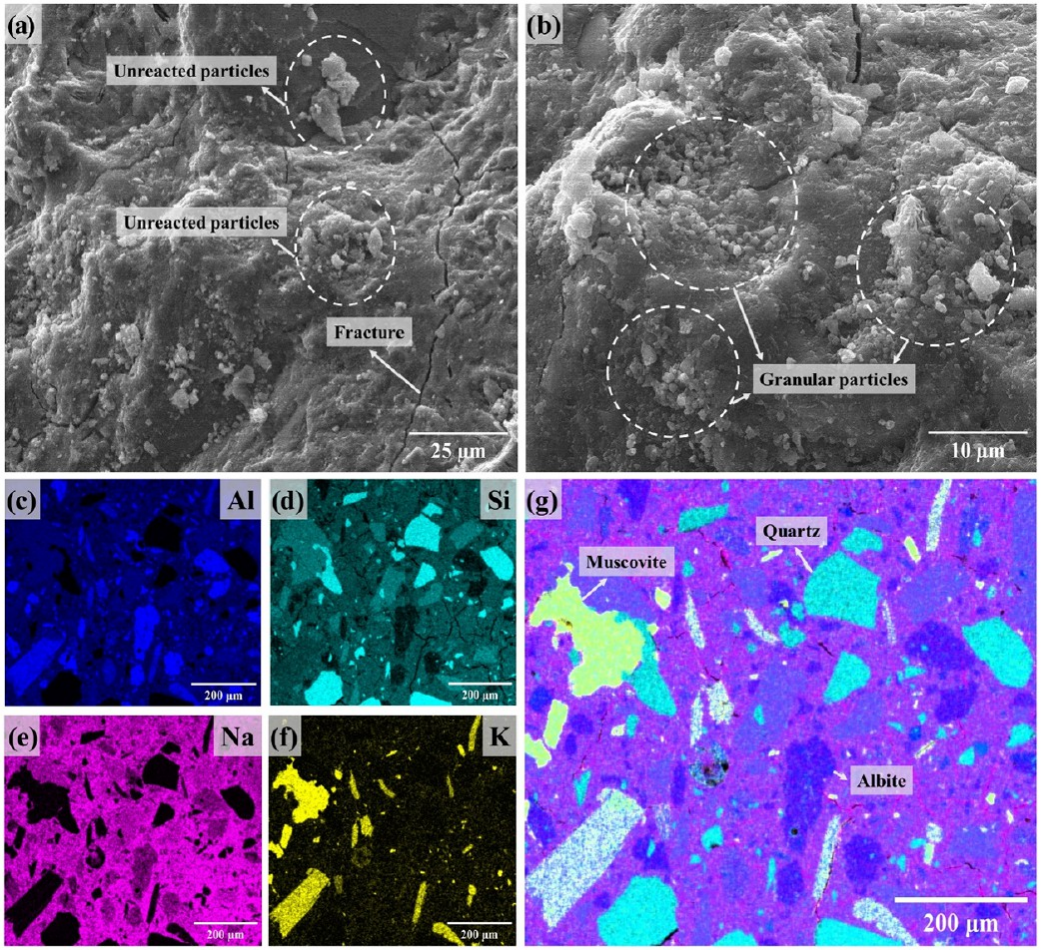
SEM images (a, b) and EDS mapping (c–g) of the geopolymer MKB.LiMW[40].

Geopolymers derived from reaction mixtures with a high content of silicate solutions exhibit a homogeneous surface, with fewer pores and a hardened microstructure.^94,95^ When comparing the alkaline solution content used in the geopolymer synthesis, all materials prepared with MKB had a lower amount of dissolved silica in the mixture (see Table 4). This chemical adjustment was necessary to prevent efflorescence and ensure the hydrolysis of the available alumina in this metakaolin. However, it might be associated with weaker intergranular bonding, reduced pore connectivity, and compromised mechanical properties.

The elemental mapping (Figure 18c–g) of the MKB-based geopolymer without LiMW is closely comparable to the data obtained for the material produced with MKS. The main difference is the presence of some points on the surface with higher concentrations of K and Si. The identifiable presence of Si is attributed to crystalline quartz in this metakaolin. As evidenced by the EDS analysis (Figure 19c–g), adding LiMW to the MKB-based geopolymer results in a similar distinction between the unreacted mining waste particles and the geopolymeric binder.

In summary, the microstructural and elemental mapping characterizations confirmed how significantly the geopolymer matrix can differ depending on the aluminosilicate (metakaolin) and the amount of alkaline solution used in the synthesis. They also demonstrated how effective the packing effect, resulting from the interaction between the geopolymer gel and the LiMW particles, was in enhancing the engineering properties of the materials.

## 4 Conclusion

This research has provided an in-depth investigation of the influence of the metakaolin aluminosilicate source, the incorporation of lithium mining waste as an aggregate, and the amount of the alkaline solution used in the geopolymer mixture on compressive strength and physicochemical properties. The characterizations performed to understand the properties of the raw materials and the resulting metakaolin-based geopolymers detailed a chemical description for the differences observed in compressive strength between the two syntheses and demonstrated the importance of comprehending the molecular structure of the precursors to develop materials with long-term durability, resulted in the following considerations:

i. The best compressive strength results after 28 days of curing were obtained for geopolymers containing 40% of LiMW: 59.3 MPa (MKS) and 43.7 MPa (MKB). These results are comparable to or even exceed those achieved with Portland cement, highlighting the applicability of LiMW as a viable component in geopolymeric binders, especially in materials applied to the civil construction sector.
ii. Three main aspects were identified as determinants of mechanical performance: (1) the effect of reactive alumina, (2) the effect of reactive silica, and (3) the liquid-to-solid ratio.
iii. The increase in LiMW content intensified diffraction peaks associated with quartz, albite, and muscovite, confirming the presence of poorly reactive crystalline phases derived from the waste. These results suggest a low contribution of LiMW as a source of aluminosilicates in the geopolymerization reaction.
iv. FT-IR spectroscopy confirmed the formation of geopolymeric materials in both syntheses, indicating that LiMW incorporation did not compromise the geopolymerization reaction. However, an intensification of the bands attributed to aluminosilicate groups from the waste was observed as its proportion increased.
v. ^27^Al NMR spectroscopy revealed predominant resonance around 60 ppm, attributed to tetrahedrally coordinated aluminum [Al­(IV)], evidencing the formation of the three-dimensional network characteristic of geopolymers, regardless of the addition of up to 40% LiMW.
vi. ^29^Si NMR spectra, deconvolutions, and quantitative analysis demonstrated different molecular arrangements of Q*^n^* (*m*Al) units, with the highest degree of condensation (Q^4^) observed in MKS-based synthesis, possibly explaining their superior compressive strength. The presence of unreactive quartz derived from the waste was also confirmed.
vii. SEM and EDS analyses exhibited a direct correlation between the chemical composition of the precursors, the alkaline solution, and the development of dense and homogeneous microstructures. LiMW acted as a filler, reducing porosity and increasing density, which positively contributed to the final mechanical performance of the geopolymers.

This work also reinforces the relevance of proactive research aligned with the United Nations Sustainable Development Goals (SDGs), focusing on reducing the CO_2_ footprint, promoting sustainable construction practices, minimizing waste through recycling and reuse, and improving the environmental management of mining tailings disposal.

## Supplementary Material



## Notes

Additional information for the adsorption analysis, including isotherms of the two metakaolin sources used in this work (PDF)
