# Supplementary material for: Synthesis and Characterization of Lithium Mining Waste and Metakaolin-Based Geopolymers
Source: ACS Omega. 2026 May 6;11(19):27913–30. doi: 10.1021/acsomega.5c11473 (PMC13191674; doi:10.1021/acsomega.5c11473)
Supplement: Supplementary file 1 [file ao5c11473_si_001.pdf]

SUPPLEMENTARY MATERIAL

Synthesis and characterization of lithium mining waste and metakaolin-based geopolymers

Vinícius F.C. Sampaio[a]*, Ana Gabriela Fernandes, Jeniffer Fernandes[a], Suellen Almeida[a], Lucas Lorenzini[a], Rochel Montero Lago[a], and Ana Paula C. Teixeira[a]*

[a] Departamento de Química, ICEx, Universidade Federal de Minas Gerais (UFMG), Av. Antônio Carlos, 6627, Pampulha, Belo Horizonte - MG, Brazil

**anapct@ufmg.br*

1. Supporting information for topic 2.1: *Raw materials*

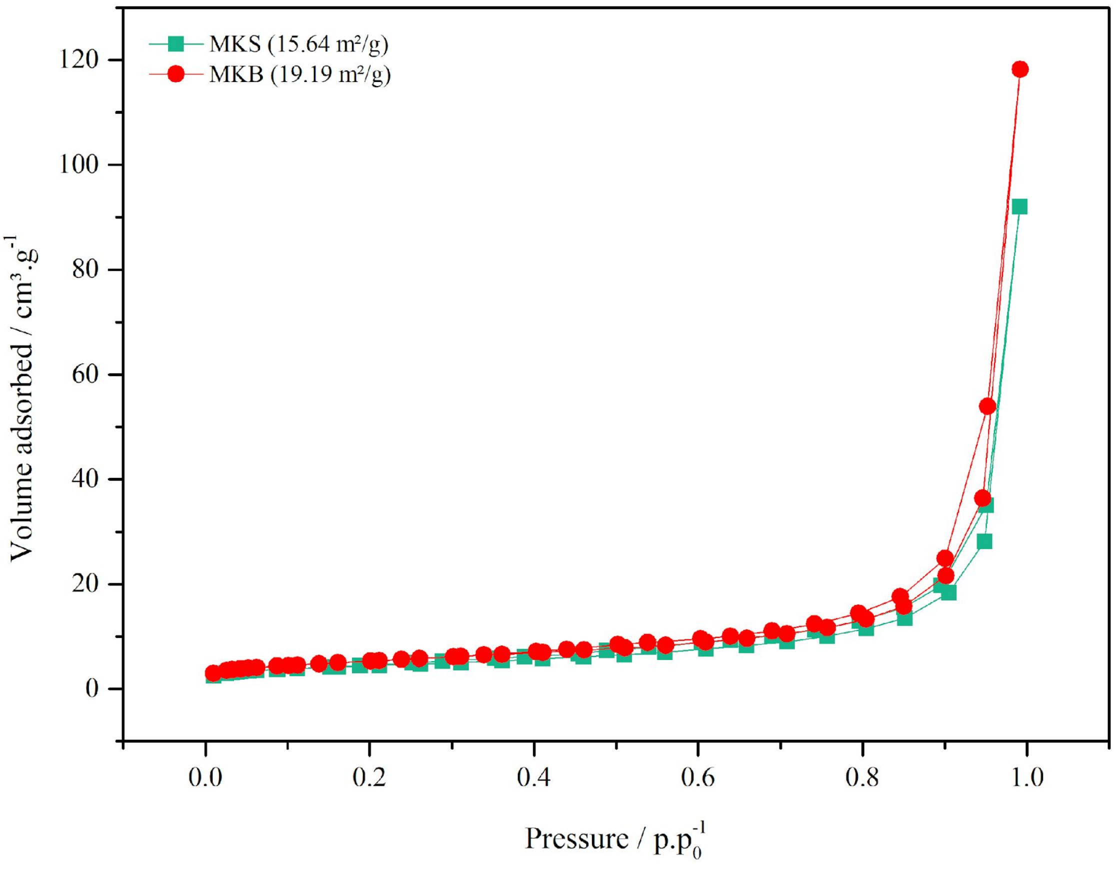

Fig.S1. N_2 physisorption isotherms of the metakaolins MKS and MKB.
